# ARCA: the interactive database for arbovirus reported cases in the Americas

**DOI:** 10.1186/s12859-023-05433-7

**Published:** 2023-08-16

**Authors:** Maria V. Meneses, Alberto Riva, Marco Salemi, Carla Mavian

**Affiliations:** 1 Emerging Pathogens Institute, University of Florida, Gainesville, USA; 2 Interdisciplinary Center for Biotechnology Research, University of Florida, Gainesville, USA; 3 Department of Pathology, Immunology and Laboratory Medicine, College of Medicine, University of Florida, Gainesville, USA

**Keywords:** Database, Arbovirus, Zika, Dengue, Chikungunya

## Abstract

**Background:**

Accurate case report data are essential to understand arbovirus dynamics, including spread and evolution of arboviruses such as Zika, dengue and chikungunya viruses. Giving the multi-country nature of arbovirus epidemics in the Americas, these data are not often accessible or are reported at different time scales (weekly, monthly) from different sources.

**Results:**

We developed a publicly available and user-friendly database for arboviral case data in the Americas: ARCA. ARCA is a relational database that is hosted on the ARCA website. Users can interact with the database through the website by submitting queries through the website, which generates displays results and allows users to download these results in different, convenient file formats. Users can choose to view arboviral case data through a table which containscontaining the number of cases for a particular week, a plot, or through a map.

**Conclusion:**

Our ARCA database is a useful tool for arboviral epidemiology research allowing for complex queries, data visualization, integration, and formatting.

## Background

Arboviruses are transmitted by blood feeding arthropods such as mosquitoes or ticks [1]. Through what is known as the sylvatic cycle, an arthropod infection occurs by ingesting blood meal from an infected vertebrate host, after which the virus begins to multiply within the arthropod’s tissues and salivary glands and can eventually be transmitted to another vertebrate host [1]. Arboviruses can infect a wide range of avian and mammalian hosts [1, 2]. Many of these hosts, however, show no clinical signs of infection, which not only allows arboviruses to thrive in nature through their sylvatic cycle [3], but also makes it difficult to detect and monitor natural reservoirs. Unknown arboviral reservoirs pose a significant threat to public health, in particular with increase in deforestation, hunting, agriculture, or urbanization leading to additional human/mosquitoes and unknown arboviral reservoirs contacts [3]. Moreover, it has been shown that forest anthropophilic mosquitos may move into areas of human habitation to feed and, thus, transmit new arboviruses [3]. In both scenarios, after humans infected with an arbovirus enter urban environments, arbovirus infections spread rapidly to other humans by urban-adapted vectors through spillover events where the sylvatic transmission cycle has “spilled over” into an urban transmission cycle [3]. As a result, an increasing number of spillover events have been identified over the past two decades, giving rise to local outbreaks as well as global epidemics (pandemics), such as like the large Zika virus (ZIKV) epidemic that occurred in the Americas in 2015–2016. During the 2015–2016 Zika epidemic, nearly 4 million cases of dengue (DENV) and chikungunya (CHIKV) virus were also reported in the American continent. Both viral diseases share the same vector with ZIKV, Aedes aegypti, indicating the co-circulation of at least three different arboviruses at the same time in the same geographic area [4]. Because these diseases have similar symptomology while their clinical presentation can vary from person to person in terms of symptoms and severity, differential diagnosis can be difficult [4], and it is possible that numerous ZIKV cases were, in fact, misdiagnosed as DENV or CHIKV, or vice versa [4].

Vector-borne diseases cause more than 700,000 deaths annually [5]. Due to socio-ecological changes and globalization, vector-borne disease dynamics are expected to evolve, becoming even more complex and multifaceted [6]. Case report data are essential to understand the pathogen spread as they reflect, in the absence of case reporting bias, the dynamic of epidemic/outbreak spread [7]. In the case of arbovirus case reporting in the Americas, this data is often inaccessible or presents discrepancies due to its different sources [8, 9]. Moreover, the use of country-specific formats of reported cases further complicates data homogenization. Currently, the database hosted by Pan-American Health Organization (PAHO) [10–12] is the main publicly accessible source of arbovirus cases reported by health ministries in the Americas. While being a great resource collecting case data from different sources, its website interface is less user-friendly for data export or reformatting. To overcome these limitations, we developed the ARCA (arbovirus reported cases in the Americas) database. ARCA is accessible to the public and stores weekly Zika, dengue, and chikungunya cases reported in the Americas. ARCA is a useful tool for arboviral epidemiology research as it provides a user-friendly interface allowing for complex queries (data mining), as well as data visualization, integration, and formatting.

## Construction and content

**Fig. 1:**
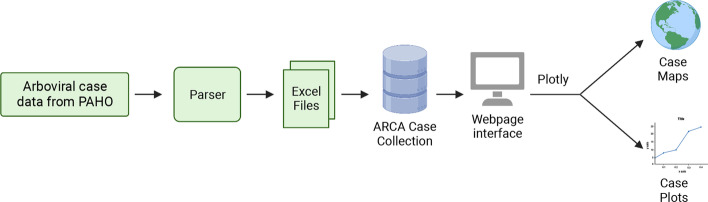
Overview of the ARCA Database Workflow. The end-to-end data ARCA workflow includes the source of data, extraction, processing, storage, and interactive visualization

ARCA is structured as a relational database using sqlite (https://www.sqlite.org/) (Fig. 1). The database is hosted on the website https://salemilab.epi.ufl.edu/ARCA/ (Fig. 1). The site was designed using a CSS library provided by w3schools.com (https://w3schools.com/w3css/), and site functionality was implemented through Python scripts. The main source for ARCA is the PAHO database for cases of Zika, dengue, and chikungunya viruses. According to what is provided by the PAHO, cases for chikungunya and zika featured in ARCA are reports of all autochthonous suspected and confirmed cases. In respect to dengue cases, these are reports of all dengue cases; suspected, probable, confirmed, non-severe and severe cases, and deaths. The confirmed cases are laboratory confirmed cases in which suspected or probable cases are confirmed with a positive test result. A parser was written in Python to extract the case-reported data from the exported Excel sheets from the PAHO database (Fig. 1). Since the PAHO database reports the cumulative cases for each virus, our parser stores the cases of the prior week and calculates the difference between the new total cases and the stored total cases from the week prior. When the cumulative cases of the prior week are greater than the cumulative cases of the current week, the parser would process that as negative case for that current week. For example, in the first week of 2018, there were 16 Zika cases in Colombia. As the year progresses, the cumulative cases continued to increase. In the 37th week of 2018 there were 703 cumulative Zika cases, but then in the following week, there were 702 cumulative Zika cases, leading to the appearance of a negative case for week 38, which would indicate a problem in case reporting. To mitigate this issue, we have implemented a strategy to maintain the highest recorded cumulative cases as the reference point (in this instance, the cumulative cases reported for week 37). Subsequently, we only record cases for weeks that surpass this reference point, while weeks with a cumulative total less than the reference point are not displayed in the table visualization. When plotting the data, we treat these intervening weeks as having zero reported cases, to ensure a consistent and coherent representation.

**Fig. 2:**
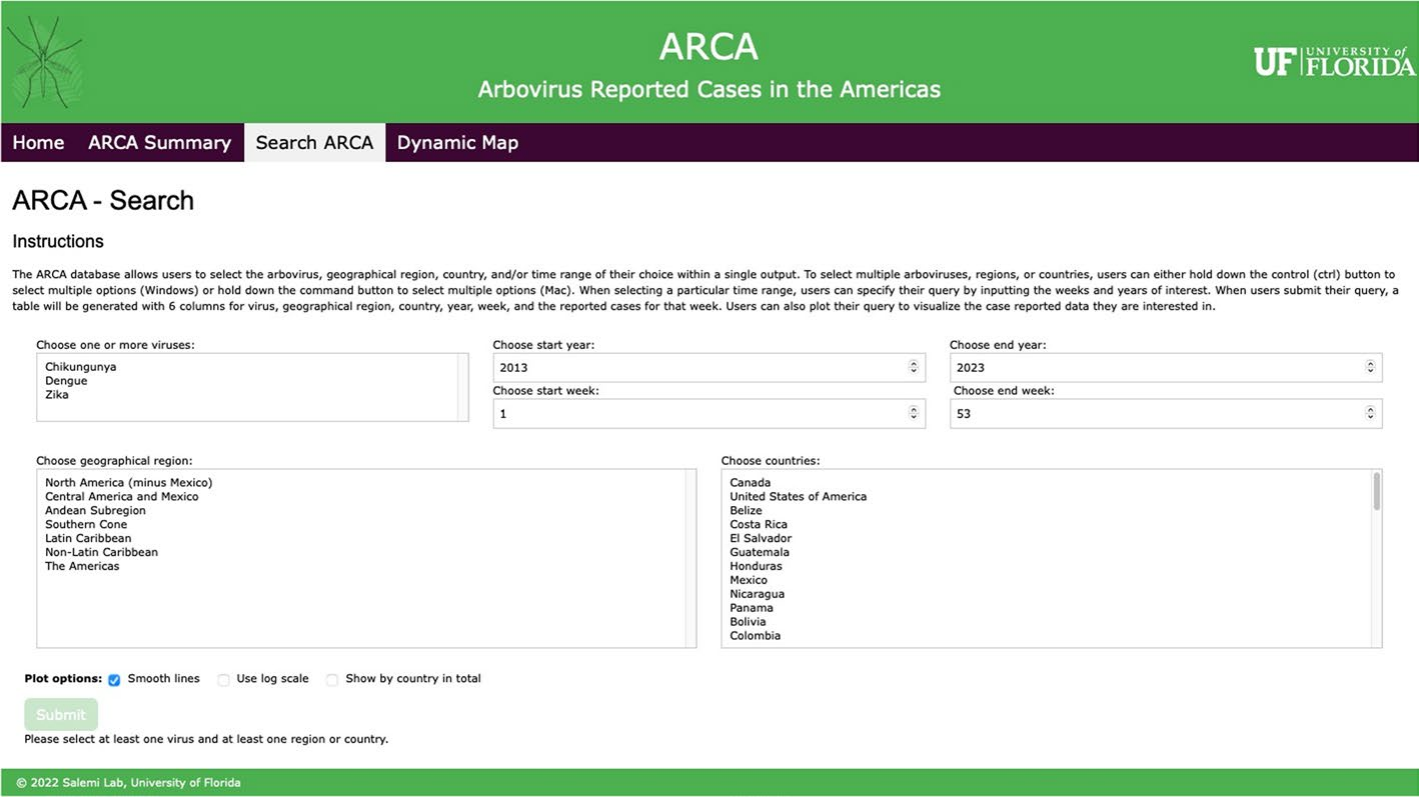
*Search page* of the ARCA database website. Users are able to make targeted queries based on virus type, time range, region, and/or country of interest. This interface enables users to retrieve relevant data efficiently based on their search criteria

**Fig. 3:**
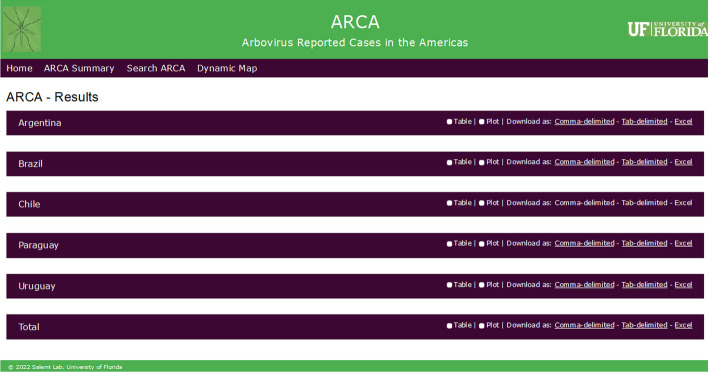
*Result page* of the ARCA database website. The *result page* displays query-based data visualization and export options. The results shown here as an example correspond to a query for the Southern Cone region and all the viruses. Each menu bar represents a country within the Southern Cone, and the final menu bar is the total of all the cases reported within this region

The ARCA website is organized into five pages: (a) the *homepage*, (b) *summary page*, (c) *search page*, (d) *results page*, and (e) *dynamic map page* (Fig. 2). (a) The *homepage* greets users with an overview of the contents within ARCA along with an explanation for each page on the menu bar. (b) *The summary page* provides an outline of the cases reported within the Americas by virus and by geographical region. The case summary table displays the time interval (in years) for which data (case reports) are available in the database for each virus. (c) The *search page* contains seven fields that users can utilize when making their query. These fields are automatically populated by the contents within the database. There is one field to choose one or more viruses available in the database, four fields to specify the time range of interest (two for the week and two for the year), one field to choose one or more geographical regions, and another field to choose one or more countries of interest. The submit button at the bottom of the webpage is disabled until a user specifies at least one virus and one country or a geographical region. If a user does not specify a time range, the database will generate all the case report data it contains for that query. Users can also control the appearance of plots that are generated in the *results page* by changing the y-axis to a logarithmic scale and enabling or disabling line smoothing for better visualization. (d) When users submit their queries, they are taken to the *results page*. A menu bar is generated for each country within the query (Fig. 3). Using each country’s menu bar, the user can choose to see a table containing three to five columns, depending on how many viruses were selected in the query. The columns correspond to the year, week, and the number of cases for each virus. When PAHO does not report cases or confirms that there were no cases in a certain country for a certain week (empty entry in PAHO table), ARCA displays “Not Reported (NR)” instead of an empty entry.

**Fig. 4:**
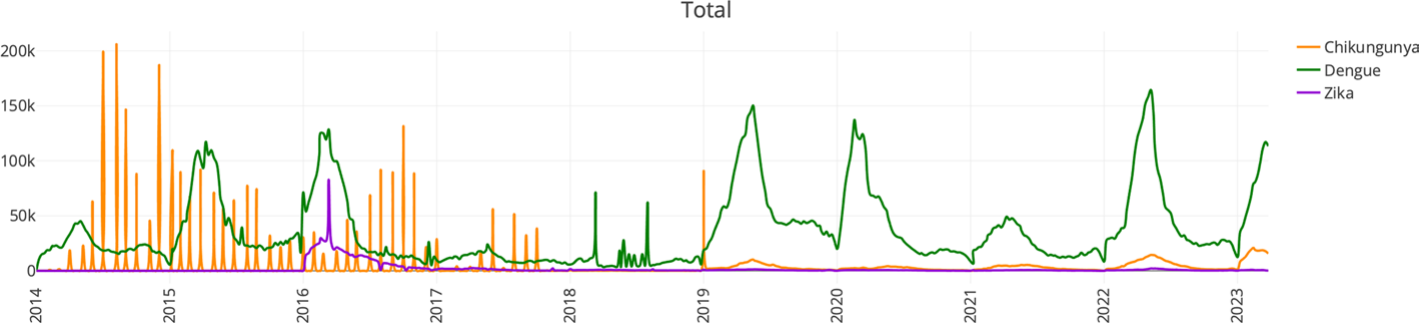
“Total” menu bar function in the *results page*. This plot demonstrates the sum of the weekly reported cases of chikungunya, dengue, and Zika respectively across all the countries in the Americas over time

**Fig. 5:**
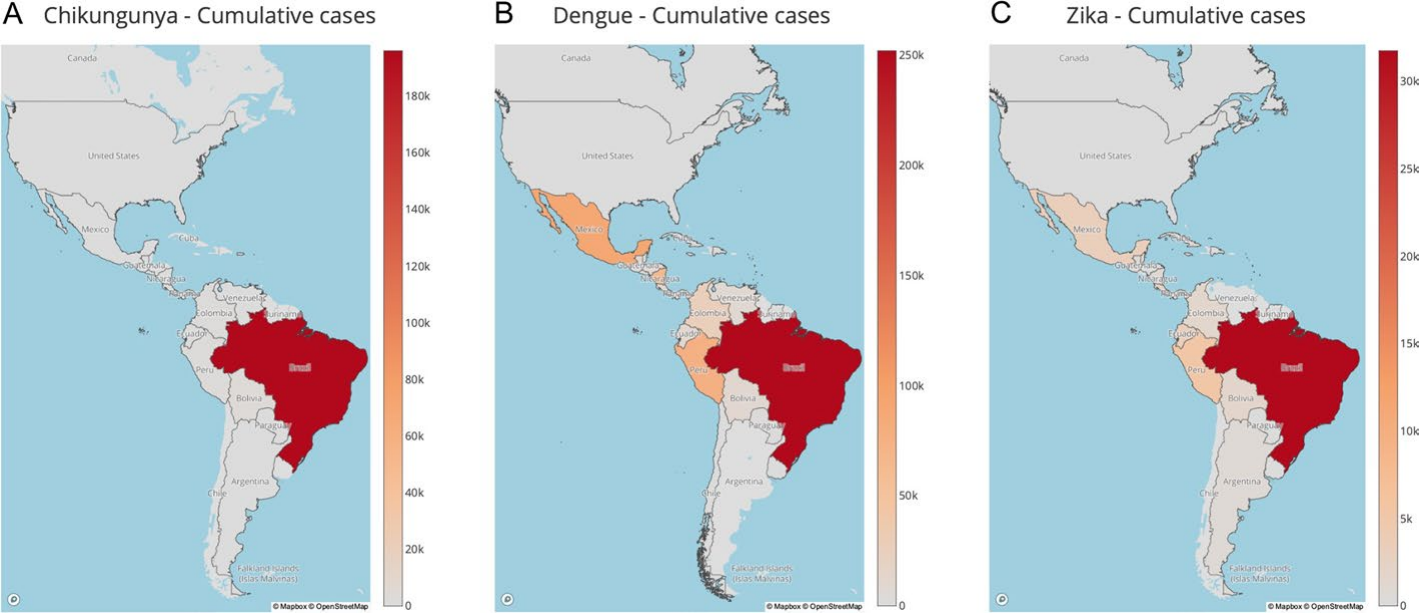
*Dynamic map page* visualizations of cumulative cases. Cumulative cases of Zika (**A**), dengue (**B**), and chikungunya (**C**) at the end of 2017. Each map illustrates the distribution and intensity of the respective viruses’ reported cases, offering insight into the prevalence and spread of these arboviruses

Users also have the option to visualize their results as a plot (generated using the Plotly JavaScript library) (Fig. 4). The plot, that can be downloaded as an SVG file, displays the number of cases reported over time in a line graph, and can show data for more than one virus. Users also have the option to download the tables containing the case-reported data for each country as comma-delimited (CSV), tab-delimited, and Excel files. Finally, if more than one country was selected in a query, an additional menu bar displays the total cases of the countries or geographical region selected. (e) The *dynamic map page*, developed using Plotly and the Mapbox library (https://www.mapbox.com/), allows users to visualize the progression of cases for all reporting countries in the Americas, by new cases or cumulatively, in a map for a particular year and virus (Fig. 5A–C). ARCA has enhanced this feature from the PAHO database as PAHO provides these maps for select viruses, years, and countries. The code for the database, the parser, and the webpage interface are freely available at https://github.com/salemilab/ARCA/

## Utility and discussion

One of the biggest obstacles when studying case reported data is the lack of data homogenization as public health organizations across the globe utilize different formats and have different standards to report cases locally. Therefore, publicly available databases like PAHO that collect arboviral cases across countries in a single source has proven to be useful. Despite this, PAHO has some usability limitations. Users are unable to submit complex queries as they can only view cases on a virus and weekly basis. In addition, data cannot be reformatted or exported to the needs of the user. Although PAHO contains data visualization tools, this feature is only available for select viruses and countries. PAHO does not provide an automatic way to extract the data from the database, therefore the only manual step in ARCA is when PAHO cases are downloaded as Excel files from the PAHO database. The parser then processes the Excel files and updates the SQLite database, which updates automatically the ARCA webpage interface.

The absence of uniform case reporting, likely due to irregular reporting by certain countries, as for example for Cuba, is to be acknowledged, especially for modelers who need uniform numeric time-series, as this limits the use of the case data provided in ARCA, or PAHO. Non uniform case reporting occurs in the PAHO database in two ways: the occasional display of a higher cumulative case reported entry for the prior week and a lower cumulative case reported entry for the subsequent week, which is not possible since the cumulative cases of subsequent weeks build from this prior cumulative case reports; and an empty entry for weeks when countries, which could be interpreted as absence of reporting. The PAHO database does not provide a reason for these occurrences. In the first instance, ARCA avoids reporting negative cases for that week, which would occur since ARCA reporting takes in account the cumulative cases of subsequent weeks build from this lower cumulative case report, and instead reports a zero; while for the second one uses the notation “NR” for weeks with no reporting. We acknowledge that our approach may not be ideal since it may overlook the actual case dynamics occurring during those weeks. However, given the lack of available information to address this issue, we believe this approach represents the best available solution.

PAHO and ARCA maps share certain features, although the characteristics differ across different arbovirus maps in the PAHO database. For instance, PAHO generates yearly cumulative maps for dengue cases, while Zika and chikungunya maps allow weekly visualization of case accumulation. The process of reaching the maps is also different: dengue maps can be found by clicking a globe icon, while chikungunya and Zika maps are available on a separate tab. Additionally, the presentation of cases differs among the arboviruses, with standardized categories based on case numbers being used for chikungunya and Zika, whereas dengue maps do not use any form of standardization. Considering these factors, ARCA offers the advantage of standardizing data communication, facilitating map access, and allowing users to have the option to visualize cases on both cumulative and weekly bases.

## Conclusions

ARCA is a key tool to study seasonality of arboviral outbreaks supporting ecological/evolutionary investigations of driving factors for epidemic spread. Currently, ARCA is the only publicly available database for arbovirus case reported data that allows users to define and filter queries, visualize, and export their results in convenient file formats within one user-friendly interface. An issue we encountered and corrected was the occasional display of a negative number of cases, due to the fact that in some cases the cumulative total for a week is lower than that of the previous week. These situations, that may be due to errors by local public health reporting agencies, are detected and handled correctly by our parser, providing an additional benefit to ARCA users. In respect to data curation, ARCA will continually be updated with newly reported cases for Zika, dengue, and chikungunya and potentially extended by including additional newly emerging or re-emerging arboviruses.

## Data Availability

The data featured in the database are available on both the summary page of the ARCA website (https://salemilab.epi.ufl.edu/ARCA/index.cgi?pg=summary) and in the ARCA Github repository (https://github.com/salemilab/ARCA.git) under the db directory. ARCA is freely accessible through the https://salemilab.epi.ufl.edu/ARCA/ website. The source code is available under an MIT license at https://github.com/salemilab/ARCA.git.

## Abbreviations

PAHO: Pan-American Health Organization
ARCA: Arbovirus reported cases in the Americas
